# Supplementary material for: Extent of Cardiac Damage and Mortality in Patients Undergoing Transcatheter Aortic Valve Implantation
Source: J Clin Med. 2021 Sep 30;10(19):4563. doi: 10.3390/jcm10194563 (PMC8509290; doi:10.3390/jcm10194563)
Supplement: Supplementary file 1 [file jcm-10-04563-s001.zip › jcm-1388706-supplementary.pdf]

Journal of
Clinical Medicine

MDPI

Extent of cardiac damage and mortality in patients undergoing transcatheter aortic valve implantation

Supplemental Material

TABLE OF CONTENTS

- Supplemental Table S1. Prevalence of cardiac damage stages and their individual components in study population 2
- Supplemental Table S2. Procedural data 3
- Supplemental Table S3. Echocardiographic parameters according to stage of cardiac damage at baseline, discharge and 30-day after TAVI 4
- Supplemental Table S4. Multivariable model 4
- Supplemental Figure S1. Staging of cardiac damage in patients with aortic stenosis 5

Table S1. Prevalence of cardiac damage stages and their individual components in study population.

**Stages of Cardiac Damage**	
Stage 0 No extravalvular cardiac damage	4/262 (1.4%)
Stage 1 Left ventricular damage	19/262 (7.2%)
Stage 2 Left atrial or mitral valve damage	106/262 (40.4%)
Stage 3 Pulmonary vasculature or tricuspid valve damage	59/262 (22.7%)
Stage 4 Right ventricular damage	74/262 (28.3%)
Individual component of stages of cardiac damage	
STAGE 0	
STAGE 1	255/262 (97.4%)
LV hypertrophy (LV mass index > 95 g/m^2 for women, >115 g/m^2 for men)	228/262 (87%)
LVEF <50%	57/262 (21.8%)
GLS <-20%	140/262 (53.5%)
E/e′ ratio >14	154/262 (58.8%)
STAGE 2	234/262 (89.3%)
Left atrial volume index (LAVI) >34 mL/	227/262 (86.6%)
Moderate/severe mitral valve regurgitation	118/262 (45%)
Atrial fibrillation	67/262 (25.6%)
STAGE 3	120/262 (45.5%)
Systolic pulmonary artery pressure ≥60 mmHg	43/262 (16%)
Moderate/severe tricuspid valve regurgitation	106/262 (40.5%)
STAGE 4	74/262 (28.3%)
Tricuspid annular plane systolic excursion (TAPSE) < 17 mm	56/262 (21.4%)
Right ventricular basal diameter (RVBD) > 42 mm	33/262 (12.6%)

Table S2. Procedural data.

	N = 262	Stage 0/1 (n = 23)	Stage 2 (n = 106)	Stage 3 (n = 59)	Stage 4 (n = 74)
Indication to TAVI					
High-flow, high-gradient symptomatic AS	246 (93.8%)	21 (91.3%)	102 (96.2%)	58 (98.3%)	65 (87.8%)
Low-flow low-gradient AS	16 (6.2%)	2 (8.7%)	4 (3.8%)	1 (1.7%)	9 (12.2%)
Access site					
Percutaneous transfemoral	255 (97.3%)	22 (95.6%)	104 (98.1%)	57 (96.6%)	72 (97.3%)
Surgical transfemoral	3 (1.2%)	1 (4.4%)	0	0	2 (2.7%)
Other	4 (1.5%)	0	2 (1.9%)	2 (3.4%)	0
Valve prosthesis					
Medtronic Evolut R	147 (56.1%)	12 (52.2%)	61 (57.5%)	39 (66.1%)	35 (47.3%)
Medtronic Corevalve	45 (17.2%)	3 (13%)	11 (10.4%)	12 (20.3%)	19 (25.7%)
Edwards Sapien 3	49 (18.7%)	7 (30.4%)	22 (20.8%)	6 (10.2%)	14 (18.9%)
Edwards Sapien XT	2 (0.8%)	1 (4.4%)	0	0	1 (1.3%)
Symetis Acurate Neo	14 (5.3%)	0	9 (8.5%)	11 (1.7%)	4 (5.4%)
St Jude Portico	3 (1.1%)	0	2 (1.9%)	1 (1.7%)	0
Other	2 (0.8%)	0	1 (0.9%)	0	1 (1.3%)
Procedural details					
Ballon aortic valvuloplastic	82 (31.3%)	6 (26.1%)	32 (30.2%)	12 (20.3%)	32 (43.2%)
Post-dilatation	26 (9.9%)	2 (8.7%)	8 (7.5%)	7 (11.9%)	9 (12.2%)
Rapid pacing	133 (50.7%)	14 (60.9%)	53 (50%)	26 (44.1%)	40 (54.0%)
Procedural time (min)	105.93 ± 47.8	115.6 ± 43.0	102.0 ± 40.2	107.6 ± 103.3	107.2 ± 30.7
Fluoroscopy time (min)	29.86 ± 13.7	30.3 ± 11.7	29.5 ± 11.9	29.9 ± 16.5	30.2 ± 10.7
Contrast volume (ml)	236.1 ± 113.6	239.3 ± 98.5	236.8 ± 126.7	245.2 ± 123.2	226.9 ± 109
Concomitant procedures					
Percutaneous coronary intervention (PCI)	24 (9.2%)	1 (4.4%)	11 (10.4%)	5 (8.5%)	7 (9.5%)
Other procedures	15 (5.7%)	1 (4.4%)	7 (6.6%)	4 (6.8%)	3 (4.0%)
Procedural success (VARC2)	258 (98.5%)	23 (100%)	105 (99%)	59 (100%)	71 (95.9%)
Complications	18 (6.8%)	1 (4.4%)	8 (7.5%)	5 (8.5%)	4 (5.4%)
Death	2 (0.8%)	0	1 (0.9%)	0	1 (1.3%)
Conversion to cardiac surgery	2 (0.8%)	0	2 (1.9%)	0	0
Ventricular wall perforation	2 (0.8%)	0	1 (0.9%)	0	1 (1.3%)
Mitral valve dysfunction	1 (0.4%)	0	1 (0.9%)	0	0
Cardiac tamponade	4 (1.5%)	0	2 (1.9%)	1 (1.7%)	1 (1.3%)
Valve migration	0 (0%)	0	0	0	0
Valve embolization	1 (0.4%)	0	0	0	1 (1.3%)
Transitory atrioventricular block	7 (2.7%)	1 (4.4%)	3 (2.8%)	22 (3.4%)	1 (1.3%)
Other	7 (2.7%)	1(4.4%)	1 (0.9%)	2 (3.4%)	3 (4.0%)
ICU length of stay	3.5 ± 2.4	3.5 ± 2.8	3.6 ± 4.7	3.7 ± 2.9	3.6 ± 2.9
Red blood cell transfusion	69 (26.3%)	3 (13%)	26 (24.5%)	14 (23.7%)	26 (35.1%)

Table S3. Echocardiographic parameters according to stage of cardiac damage at baseline, discharge and 30-day after TAVI.

	Stage 0/1 (n = 23)	Stage 2 (n =106)	Stage 3 (n = 59)	Stage 4 (n = 74)	p-Value
Baseline					
LVEF, %	62.6 ± 7.9	58 ± 7.7	54 ± 13.2	50.7 ± 13.1	0.003
LVGLS, %	19.1 ± 1.6	17.5 ± 2.7	17 ± 4	17 ± 4.3	0.329
E/E'ratio	13.3 ± 3.7	14.7 ± 5.2	17.2 ± 4.3	18.8 ± 8.5	0.006
LAVI, ml/m^2	29.2 ± 2.8	51 ± 12.8	55 ± 14.7	63.6 ± 15.2	<0.001
PAPS, mmHg	37.6 ± 8	36.9 ± 7.2	48.4 ± 12	50.1 ± 12.1	<0.001
TAPSE, mm	22.7 ± 4.3	21.3 ± 3.4	21.3 ± 4.3	17.9 ± 5.3	<0.001
Discharge					
LVEF, %	58.2 ± 6.7	56.7 ± 6.1	55.9 ± 8.7	52.4 ± 10.5	0.064
LVGLS, %	20 ± 1.1	18.6 ± 2.1	18 ± 2.8	18.2 ± 3.5	0.14
E/E'ratio	14.4 ± 4.9	15.7 ± 5.7	18 ± 5.8	18.3 ± 7.1	0.082
LAVI, mL/m^2	31.7 ± 5	47.6 ± 14	53.7 ± 17	60.7 ± 16.1	<0.001
PAPS, mmHg	36.4 ± 8.8	38.9 ± 7.8	42.6 ± 9.2	46.1 ± 12.9	0.003
TAPSE, mm	21 ± 2.4	20.7 ± 3.3	21 ± 3.1	19.3 ± 3.2	0.09
30-day					
LVEF, %	62.2 ± 4.9	59.5 ± 6.2	57.8 ± 8.3	56.3 ± 7.5	0.048
LVGLS, %	21.6 ± 1.1	20.1 ± 2.3	18.8 ± 3.6	19.8 ± 3.5	0.039
E/E'ratio	11.8 ± 2.2	12.9 ± 4.5	13.9 ± 4.9	14.4 ± 4.2	0.24
LAVI, mL/m^2	29.4 ± 3.7	42.6 ± 14	49.8 ± 16	55.4 ± 14.6	<0.001
PAPS, mmHg	31.8 ± 5.1	34.3 ± 5.4	41.2 ± 11	40.8 ± 11.1	<0.001
TAPSE, mm	23.6 ± 3.1	22.1 ± 3	21.9 ± 3.1	21.6 ± 3	0.259

Table S4. Multivariable model.

	Hazard Ratio	Standard Error	95% Confidence Intervals	*p*-Value
Stage of cardiac damage	1.75	0.35	1.17–2.60	0.006
NYHA class	1.73	0.67	0.81–3.71	0.159
Fraility	6.16	3.90	1.77–21.37	0.004
STS score	1.04	0.03	0.97–1.11	0.231
Hypertension	2.58	6.55	0	1.0
Dylipidemia	1.53	0.60	0.71–3.31	0.283

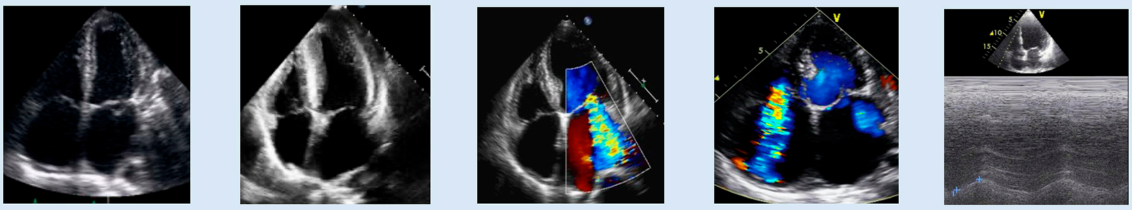

Stage 0	Stage 1	Stage 2	Stage 3	Stage 4
No cardiac damage	Left ventricular damage	Left atrial or mitral damage	Pulmonary vasculature or tricuspid damage	Right ventricular damage
	• LV hypertrophy: LV mass index >95 g/m² ♀, >115 g/m² ♂<br>• Severe LV diastolic dysfunction (E/e' >14)<br>• LV systolic dysfunction (LV ejection fraction <50%)<br>• LVGLS <20%	• LA enlargement (LA volume > 34 ml/m²)<br>• Presence of atrial fibrillation<br>• Moderate-severe mitral regurgitation	• Systolic pulmonary arterial pressure ≥ 60 mmHg<br>• Moderate/severe tricuspid valve regurgitation	• TAPSE < 17 mm

Figure S1. Staging of cardiac damage in patients with aortic stenosis.
